# Deconstructing Syndemics: The Many Layers of Clustering Multi-Comorbidities in People Living with HIV

**DOI:** 10.3390/ijerph17134704

**Published:** 2020-06-30

**Authors:** Emmanuel Peprah, Elisabet Caler, Anya Snyder, Fassil Ketema

**Affiliations:** 1 Department of Social and Behavioral Sciences, School of Global Public Health, New York University, New York, NY 10003, USA; as13565@nyu.edu; 2 Office of AIDS Research, National Institutes of Health, Bethesda, MD 20852 USA; lis.caler@nih.gov; 3 National Heart, Lung, and Blood Institute, National Institutes of Health, Bethesda, MD 20892, USA; fassil.ketema@nih.gov

**Keywords:** HIV, Syndemics, non-communicable diseases, public health, communicable diseases, cardiovascular disease, lung diseases, low and middle-income countries, sleep disorder, health disparity, comorbidity

## Abstract

The HIV epidemic has dramatically changed over the past 30 years; there are now fewer newly infected people (especially children), fewer AIDS-related deaths, and more people with HIV (PWH) receiving treatment. However, the HIV epidemic is far from over. Despite the tremendous advances in anti-retroviral therapies (ART) and the implementation of ART regimens, HIV incidence (number of new infections over a defined period of time) and prevalence (the burden of HIV infection) in certain regions of the world and socio-economic groups are still on the rise. HIV continues to disproportionally affect highly marginalized populations that constitute higher-risk and stigmatized groups, underserved and/or neglected populations. In addition, it is not uncommon for PWH to suffer enhanced debilitating conditions resulting from the synergistic interactions of both communicable diseases (CDs) and non-communicable diseases (NCDs). While research utilizing only a comorbidities framework has advanced our understanding of the biological settings of the co-occurring conditions from a molecular and mechanistic view, harmful interactions between comorbidities are often overlooked, particularly under adverse socio-economical and behavioral circumstances, likely prompting disease clustering in PWH. Synergistic epidemics (syndemics) research aims to capture these understudied interactions: the mainly non-biological aspects that are central to interpret disease clustering in the comorbidities/multi-morbidities only framework. Connecting population-level clustering of social and health problems through syndemic interventions has proved to be a critical knowledge gap that will need to be addressed in order to improve prevention and care strategies and bring us a step closer to ending the HIV epidemic.

## 1. Syndemics of HIV/AIDS

People with HIV/AIDS (PWH) suffer from interactions of both communicable and non-communicable diseases. In the context of non-communicable diseases (NCD), cardiovascular comorbid conditions such as hypertension, myocardial infarction, and atherosclerosis are increasingly prevalent in PWH who are treated with antiretroviral therapy (ART) globally. HIV-associated cardiomyopathy and pulmonary hypertension are highly prevalent within PWH in low-and middle-income countries (LMIC) [1]. Additionally, sleep disturbances are prevalent in PWH along with the increased risk for other comorbid disorders such as cardiovascular disease (CVD), hypertension (HTN), stroke, and diabetes which lead to the syndemic emergence of diseases in PWH in the United States and in LMICs [2,3].

**Figure 1:**
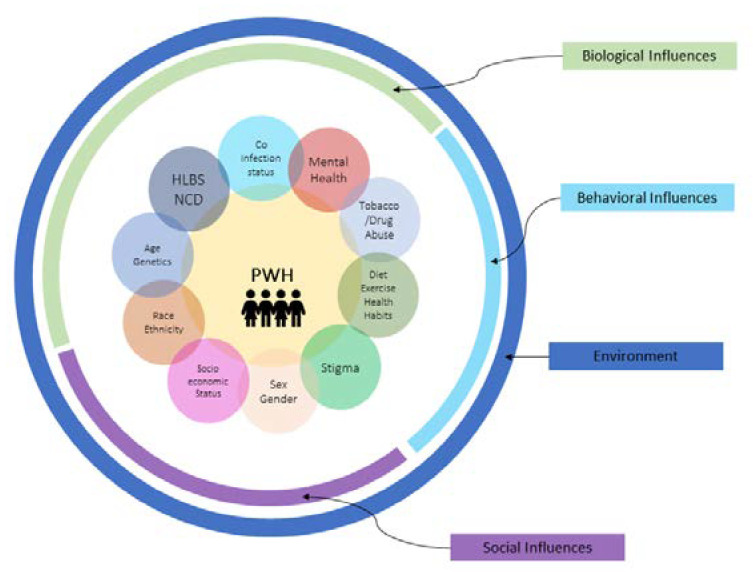
Biological, Social and Behavioral Influences that Promote Syndemic Disease Clustering in PWH.

The Framework of Syndemics (Figure 1) provides powerful strategies for recognizing how social, behavioral, political, and ecological factors create and perpetuate structural vulnerabilities that contribute to syndemic emergence (synergistic epidemic) and exacerbation of the progression of diseases in PWH. Moreover, it enables the understanding of how certain individuals, families, and communities are consigned to harmful environments that make them vulnerable to syndemics with concrete effects on social and biological wellbeing [4]. Additionally, syndemic knowledge makes it possible to intervene effectively at both the policy and clinical levels by addressing both the root causes of sickness and the disparity/inequality in the treatment of symptoms (clinical care). It has been eloquently stated by Mendenhall and colleagues that “how we think about disease pathologies affects how we design policies and deliver care to those most affected by social and economic inequities. Conventional frameworks in medicine and public health, such as comorbidity and multi-morbidity, often overlook the effects of social, political, and ecological factors” [5]. In the context of PWH having a full lifespan, chronic diseases within this population around the globe are starting to emerge. Utilizing the comorbidities framework has advanced our understanding of the underpinnings of disease from a molecular and mechanistic perspective; however, this framework lacks the fundamental elements to incorporate economic, psychological, and large-scale social forces that precipitate disease clustering in marginalized populations for PWH around the globe. Based on the work of Singer, the term *synergistic epidemics* or *syndemics* was coined to capture the largely non-biological aspects of disease clustering that is lacking in the comorbidities/multi-morbidities framework [4]. Syndemics involve population-level clustering of social and health problems. The criteria of a syndemic are as follows: (1) two (or more) diseases or health conditions cluster within a specific population; (2) contextual and social factors create the conditions in which two (or more) diseases or health conditions cluster; and (3) the clustering of diseases results in adverse disease interaction, either biological or social or behavioral, increasing the health burden of affected populations [4]. Within this context, Singer cites some powerful examples of how syndemic interaction of food insecurity increases the risk of HIV transmission by promoting involvement in risky behaviors (such as commercial sex activities) and worsens HIV clinical outcomes [4]; moreover, mental health and the psychosocial elements of discrimination are emerging areas of research [6]. Furthermore, for implementation research, syndemic intervention can strengthen prevention and care strategies by considering the full scope of syndemic vulnerabilities, rather than treating disorders individually and ignoring the complex contexts in which they occur [4].

## 2. Co-Occurrence of HIV/AIDS with other Chronic Diseases

Of the estimated 37.9 million people living with HIV/AIDS globally in 2018, approximately 23.3 million are receiving antiretroviral therapy, and this number is expected to increase [7]. While ART improves life expectancy significantly and decreases susceptibility to HIV-related infections, it also leads to an increase in the incidence of age-related NCD such as chronic cardiovascular, pulmonary, and hematological conditions [7,8]. ART is known to increase the risk of heart failure and other cardiopulmonary conditions [9,10,11]. In sub-Saharan Africa ~23.5 million people live with HIV and the majority are on highly active antiretroviral therapy (HAART) [12,13,14]. As people live longer, the prevalence of HIV infection in LMICs and its co-occurrence with other CD and NCD are increasing compared to high-income countries (HICs). Current models predict that while HIV/AIDS will not be among the top 10 leading causes of death for high-income countries, it will remain in the top five for LMICs, and will cluster with other NCDs [14]. Countries within Central and South America and the Caribbean, Central and sub-Saharan Africa, and Southeast Asia regions have the potential to be the most negatively affected by the co-occurrence of syndromes, revealing a major health disparity and the need to address the challenges in these regions, as well as the urgency to gain a deeper understanding of the root causes of disease clustering in PWH and ways to mitigate them effectively.

## 3. HIV—Noncommunicable Disease Interactions

PWH suffer from multiple chronic diseases including CVD, chronic obstructive pulmonary disease (COPD), HTN, and clinical depression. Depression among PWH is common, with rates of major depressive disorder nearly three-fold higher than the general population [15]. Throughout history, HIV- and AIDS-related stigmas have led to discrimination and violence against individuals affected by the disease, which is especially challenging for marginalized groups including sexual minorities and injection drug users [16,17,18]. As a result, many suffer from mental health issues and are more likely to exhibit unhealthy or harmful behaviors such as overeating, smoking, drinking in excess, unsafe sexual practices and drug abuse, which may lead to other conditions/diseases in many populations [19]. Research suggests that these unhealthy behaviors, specifically smoking and drinking, increase the risk of hypertension and coronary heart disease in this already vulnerable population [20,21]. The role of substance abuse (e.g., opioids) in the clustering of cardiovascular, lung, blood, and sleep disorders/diseases in PWH is also not clearly understood. Furthermore, stigma, discrimination, and violence have inhibited many from seeking or receiving proper healthcare.

While there are several social factors associated with depression among PWH, there are also several biological factors. HIV, along with other viral infections, can cause cytokine-induced sickness, which can mimic depression with symptoms including loss of appetite, lack of motivation, changes in sleep and isolation [22]. Additionally, ART can cause mood changes, anxiety, and depression which attributes to the higher prevalence of depression among PWH. In addition, the physical ailments associated with HIV can have a negative effect on everyday mood, which can keep an individual in a state of depression, as many describe the symptoms as a constant reminder of their infection [23].

COPD is the third leading cause of death by disease in the US [24]. In comparison to people without HIV, PWH have a higher prevalence of smoking-related illness, and other pulmonary conditions, such as pulmonary hypertension, asthma, and diminished pulmonary diffusing capacity (DLCO) [25]. In addition, recent data presented at Conference on Retroviruses and Opportunistic Infections (CROI) showed that COPD increases the risk of heart attack among PWH, especially type 2 heart attacks in which the vessels in the heart contract rather than becoming blocked by a ruptured plaque [26]. In the US, currently, 11% of HIV-positive veterans also have COPD, and 2% suffer from emphysema. The management of COPD and emphysema within the US veteran population is of significance to the US Department of Veteran Affairs [27,28]. Thus, the importance of understanding the syndemic interactions of HIV and COPD with other lung diseases in the HIV+ population will be indispensable in addressing this significant problem in the US and global populations [29].

On a final note, it is extremely important to mention that, while most people who receive an HIV diagnosis in the US live in urban areas, many live in rural areas particularly in the South and the Midwest. According to CDC, 23% of new HIV diagnoses in the South are in suburban and rural areas, and in the Midwest, 21% are suburban or rural—higher proportions than in the North and West. As these communities have unique healthcare needs, ‘rural residence’ is a risk factor for lower rates of HIV testing, later HIV diagnosis, later adoption of advances in ART and, consequently, increased HIV-related mortality. In addition, rural residents with HIV infection often face challenges such as stigma, social isolation, long distances to care, limited transportation, and lack of access to providers with HIV expertise [30]. A better understanding of all the factors contributing to the increasing numbers of comorbidities in these regions is urgently needed, and research implementing syndemics frameworks will be fundamental to address this urgency.

## 4. HIV-Communicable Disease Interactions

In the context of CD coinfections, it is well described that tuberculosis (TB) and HIV act synergistically to cause excess morbidity and mortality among PWH who are more likely to develop TB because of their immunodeficiency. Of the approximately 37.9 million people living with HIV, more than one-third are also infected with TB. In fact, TB is the leading killer of people with HIV, accounting for 1 in 3 AIDS-related deaths [6]. In HIV-infected individuals, TB often occurs outside the lungs, evading traditional diagnostic tests and making people unaware that they have TB. Poorly ventilated facilities such as hospitals, homeless shelters, prisons, and clinics can promote the spread of TB to vulnerable HIV-positive individuals, further fueling the syndemic [31]. This can be especially problematic as anti-TB drugs can directly interfere with antiretroviral therapy [32]. Therefore, understanding the intersection and syndemic interaction between TB and HIV is necessary to provide adequate control of this deadly combination [33,34]. On top of the HIV/TB health challenge, the use of tobacco, while not a communicable disease, is yet another global epidemic that collectively with HIV/TB amplifies the health impacts of the individual factors. The three epidemics (HIV, TB and tobacco use) are often approached individually in low-income countries. These syndemics share common social determinants of health (poverty, low-education, high population density, cultural patterns), and health systems could benefit from holistic frameworks to integrate all factors to improve disease outcome and clinical practice.

## 5. Economic Burden of Co-Morbid CDs and NCDs for PWH

The shift in prognosis from life-threatening to a chronic but manageable disease has been a major public health achievement in the HIV/AIDS community. PWH initiating ART can now expect to have similar life expectancies as their HIV-negative counterparts, barring significant HIV-related comorbidities. However, coupled with the fact that these additional years of life require treatment for survival and that PWH are more susceptible to opportunistic infections as well as a multitude of other chronic diseases, a diagnosis of HIV now presents as a critical economic burden. Currently, the average annual cost of treating HIV/AIDS alone is approximately USD 20,000 among all levels of CD4 count [35]. A male living in Kenya diagnosed at age 20 could expect to pay a lifetime total of almost USD 1 million. It is important to consider that this value ignores the indirect costs associated with treating HIV/AIDS, such as transportation, the time and effort required of family members to assist in managing the infection, as well as the larger, contextual burden of productivity loss which has great potential to negatively affect economic stability. In Africa, economic growth can be reduced by up to 4% each year as a result of HIV/AIDS-related productivity loss [36].

Common comorbidities such as TB, CVD, COPD, HTN and depression exacerbate the economic burden for PWH, therefore an integrated model of care (Syndemic Care) that addresses relevant comorbidities and the social and behavioral factors that fuel them is likely to result in better health outcomes in the long term as well as reduce the economic burden on PWH over the course of their lives. This is especially relevant in the context of the Coronavirus 2019 (COVID-19) pandemic as discussed in a very recent (April 2020) publication on the burden of COVID-19 in PWH [37].

An integrated model that provides a continuum of care, rather than an individualized treatment plan, will ensure that comorbidities are capable of being managed and increases the probability that future comorbidities will be prevented. It has been shown that the average monthly cost of care is significantly higher among PWH co-infected with TB as opposed to those with HIV only [36]. A care plan that incorporates TB and other related comorbidities into the current treatment plan would not only reduce the individual economic burden, but the regional and even national economic burden as well. Therefore, a comprehensive model of care incorporating both CD and NCD management is desperately needed to reduce the global economic burden of HIV comorbidities especially in LMICs where resources are already scarce.

## 6. Conclusions

There is a significant opportunity for research that characterizes and integrates cardiovascular, pulmonary, and other clinical aspects of both CD and NCDs using the lens of syndemics to better comprehend the synergistic effects of these diseases in PWH while also incorporating social, behavioral, political, and ecological factors which underlie diseases. The goal should be to gain a deeper understanding of the interplay between these factors and their role in promoting disease clustering at the population level, and the impact they have on disease pathologies at the individual and community level with the intent to encourage more holistic approaches in the clinical management of PWH. Moreover, the economic impact of HIV/AIDS in LMICs suggests that a comprehensive model of care which includes syndemic characterization of various factors that underpin the epidemic in distinct regions of the world is needed. This multifaceted approach which incorporates both clinical and non-clinical elements in the management of chronic disease can provide the evidence-based need for sustaining interventions to reduce the burden of diseases among PWH.
